# Business models of public private partnerships in publicly-financed emergency response services

**DOI:** 10.1186/1753-6561-6-S5-P8

**Published:** 2012-09-28

**Authors:** T Sundararaman, Arun Nair, Tushar Mokashi, Gautam Chakraborthy

**Affiliations:** 1 National Health Systems Resource Centre, New Delhi, India

## Introduction

The last six years of India’s National Rural Health Mission (NRHM) has witnessed the emergence of a wide variety of public funded, privately managed ambulance services across the states. Today there are over 3850 government financed emergency response ambulances and another 3000 are just about to start up, whereas just five years ago there were almost none. States have chosen different models for emergency response systems with varying levels of investments and effectiveness.

## Methods

The objective of this study was to analyse the three different business models of emergency response services (ERS) that have evolved under NRHM across the country. We conducted document analysis to understand the various types of publicly-financed partnership models across the country. Based on this, three distinct business models of public-private partnership in ERS were identified. To further understand the three business models, three separate case studies in states where they are most mature in terms of design, number of years of running, continuity and efficiency of management.

The three business models we studied are (a) Dial 108; (b) Haryana Swasthya Vahan Sewa (HSVS); and (c) Janani Express. Dial 108 was studied at its most mature site, Andhra Pradesh. We chose three districts in Andhra Pradesh, each from one of the three different geographical regions: coastal, *Rayalaseema*, and *Telengana* regions. We collected qualitative data and secondary data in these three districts. HSVS model is a district model with assured referral transport for pregnancy as primary focus, and emergency response as secondary. Of the three states, which have opted for this approach (Haryana, Jharkhand and Chhattisgarh), we studied the model in the more mature and established amongst the three states, Haryana. This model was studied in three districts of Haryana, one each from north, south and west-central regions of the state. Janani express, a local partnership-based model of assured referral transport, we studied the programme in Nabrangpur, where the model had matured and developed to its best through a considerable local innovation and adaptation with a continuity of local leadership.

The study compares the strengths and weaknesses of each of these models in terms of (1) coverage, timeliness, prioritization and quality of emergency response; (2) provision of assured cashless transport for pregnant-women and sick-newborns; (3) costs and sustainability of the models; (4) equity of access to these services; and (5) outcomes with respect to the rest of the emergency healthcare chain. Based on the analysis of these models, the possible roadmap to ERS in the country and the principles of design that could be used to improve the efficiency and effectiveness of each model for the coming five year plan period is also laid down.

## Results and discussion

This study was used by the Ministry of Health and Family Welfare of the Indian government to re-define its financing policy, giving states greater ownership and risk, leading to improved financing and efficiency. We find that there is a need for a more sound financing policy, caution against a provider monopoly and note that a disproportionate attention to rescue without sufficient attention to the rest of the emergency management provision is counter-productive. Legal challenges to existing policies of procurement also referred to evidence from NHSRC studies. Subsequently, eight more states adopted a more competitive approach to procurement and financing. More new players entered the ERS market. Alternate models have also emerged. Comparison across models in the second-generation studies provided inputs into strengths and limitations of each and helped improve designs. This mechanism of evaluation and feedback is essential to ensure constant improvements in effectiveness, efficiency and governance of such a massive public health effort.

## Competing interests

None declared.

## Funding statement

The study was funded by the National Health Systems Resource Centre, New Delhi.

